# Uncommon triggers of insulin autoimmune syndrome: a case report

**DOI:** 10.1186/s13256-024-04616-x

**Published:** 2024-06-27

**Authors:** Ishanya Abeyagunawardena, Chirath Madurapperuma, Poobalasingham Thuvarakan, Zeenath Thowfeek, Nagaratnam Sujeewa, Gaya Katulanda, Harindra Karunatilake

**Affiliations:** National Hospital of Sri Lanka, Colombo, Sri Lanka

**Keywords:** Hirata disease, Omeprazole, Coriander, Ginger, Case report

## Abstract

**Background:**

Insulin autoantibody syndrome (IAS), or Hirata disease, is caused by high concentrations of insulin autoantibodies, which result in spontaneous, mainly post-prandial, hypoglycemic episodes. We report a case of a previously healthy 67-year-old man presenting with recurrent fasting hypoglycemia culminating in a diagnosis of insulin autoimmune syndrome linked to omeprazole and probably spices, namely, coriander, and ginger.

**Case presentation:**

A previously healthy 67-year-old Sinhalese man presented with recurrent syncopal attacks for 3 months, which were found to be hypoglycemic episodes. He experienced mainly fasting hypoglycemic attacks, at a frequency gradually increasing to daily attacks. His cardiovascular, respiratory, abdominal, and neurologic examinations were normal. He was found to have insulin levels > 6000 mU/L and a post-polyethylene glycol insulin recovery of less than 9.5%. Contrast-enhanced computed tomography of the pancreas was normal. The diagnosis of insulin autoantibody syndrome was confirmed by testing for the insulin autoantibody level, yielding a level of > 300 U/mL. With regard to a possible trigger, he had a history of omeprazole intake for 2 weeks, 4 weeks prior to the onset of symptoms. He also consumed an herbal supplement containing coriander and ginger extracts daily for a period of 1 year, approximately 2 years prior to the onset of hypoglycemic attacks. He was commenced on prednisolone 30 mg daily, and hypoglycemic episodes responded dramatically, and thus he was tapered off corticosteroids.

**Conclusion:**

Omeprazole-induced insulin autoantibody syndrome is likely in this patient; however, the known hypoglycemic effects of coriander and ginger make it worthwhile to consider a possible association with insulin autoantibody syndrome. In addition, this case report highlights the need to consider insulin autoantibody syndrome even in patients presenting with fasting hypoglycemic attacks.

## Background

Insulin autoantibody syndrome (IAS), or Hirata disease, is caused by high concentrations of insulin autoantibodies, which result in spontaneous hypoglycemic episodes. It is a rare condition that is thought to be triggered by drugs, viruses (mumps, measles, rubella, coxackie B virus, varicella zoster, and hepatitis C), and hematological disease [1]. Case reports of patients developing IAS without any exposure to known triggers have been reported mainly in Japan; however, it has been postulated that these cases may have been due to unrecognized triggers [2]. Currently, alpha-lipoic acid and methimazole are the well-recognized drugs that trigger IAS [1].

Generally, patients with IAS have postprandial hypoglycemia [1]. However, we report a case of previously healthy 67-year-old man presenting with recurrent fasting hypoglycemic episodes culminating in a diagnosis of IAS. Further novelty lies in the possible trigger, the over-the-counter use of omeprazole or, possibly, the use of an herbal supplement with coriander and ginger as main ingredients.

## Case presentation

A 67-year-old previously healthy Sinhalese man presented to a tertiary care center in Sri Lanka with recurrent syncopal episodes for 3 months. He had multiple episodes out of hospital resulting in falls, and these episodes were associated with sweating, with no chest pain or palpitations. A random blood glucose value done at the two most recent episodes revealed values of 52 mg/dL and 64 mg/dL. The frequency of attacks had increased to daily attacks at the time of his seeking medical attention. It was noted that the attacks occurred mainly when fasting. He had no history of smoking and was a non-alcoholic. He had a history of consuming over-the-counter omeprazole for indigestion 4 weeks prior to the onset of symptoms for a period of 2 weeks and a native herbal supplement daily to boost immunity with the ingredients *Coriandrum sativum* (coriander), *Coscinium fenestratum* (yellow vine), *Zingiber officinale* (ginger), and *Alpinia calcarata* (ginger family) for a period of 1 year, 2 years before the onset of symptoms. There was no previous medical history of note and no family history of non-communicable diseases or any other illnesses. He had good family support and no significant psychosocial stressors.

On examination, he was an averagely built male with a pulse rate of 68 beats per minute and a blood pressure of 120/70 mmHg, and the cardiovascular, respiratory, abdomen, and neurological examinations were normal.

His full blood count revealed a white blood cell (WBC) count of 5.2 × 10^3^/µL (4–11), hemoglobin of 14.2 × 10^3^/µL (11–16), and a platelet count of 192 × 10^3^/µL (150–400). His serum sodium level was 139 mmol/L (135–145), potassium was 3.9 mmol/L (3.5–5.5), and he had an aspartate aminotransferase (AST) level of 24 U/L (5–34) and ALT of 50 U/L (0–55). Electrocardiography was normal. His serum cortisol level was 345 nmol/L (101–536), thyroid stimulating hormone (TSH) was 1.0 mIU/L (0.5–4.7), and free thyroxin levels were 0.97 ng/dL (0.8–1.7). He had a hemoglobin A1c (HbA1c) of 5.4% (< 5.7). His total cholesterol level was 238 mg/dL (< 200), triglyceride level was 217 mg/dL (< 150), high-density lipoprotien (HDL) level was 60 mg/dL (> 40), low-density lipoprotein (LDL) level was 124 mg/dL (< 130), and non-HDL level was 168 mg/dL (< 130). Numerous spontaneous hypoglycemic episodes were noted during the admission with blood glucose levels of < 50 mg/dL. Table 1 summarizes his plasma glucose levels and the insulin and C-peptide levels during a hypoglycemic attack.

**Table 1:** Insulin and C-peptide levels during a hypoglycemic attack

Test	Blood glucose level	Insulin (5–15 mU/L)	C-peptide (0.5–2 ng/mL)	Insulin: C-peptide molar ratio
Fasting (pre-breakfast)	48 mg/dL	> 6000 mU/L	17.19 ng/mL	> 7.32
1-hour postprandial	98 mg/dL			
2-hour postprandial	90 mg/dL			

Endogenous hyperinsulinemia is suggestive if insulin is > 3 mU/L, C-peptide is > 0.6 ng/mL, and an insulin (pmol/L)/C-peptide ratio is < 1. Therefore, it was concluded that endogenous hyperinsulinism was unlikely; however, the possibility of insulin autoantibody syndrome needed to be excluded. Contrast-enhanced computed tomography (CECT) pancreatic protocol was performed, which was normal.

The insulin-polyethylene glycol (PEG) precipitation test was carried out, and the results are summarized in Table 2.

**Table 2:** Insulin-polyethylene glycol precipitation test results

Sample	Results
Pre-PEG insulin	> 6000 mU/L
Post-PEG insulin	578.8 mU/L
Recovery (%)	< 9.5%

The test results were interpreted according to the cut offs published in 2014 by Gullo *et al.*, namely if post-PEG recovery was 5–10%, insulin autoantibody syndrome is likely, and if > 70%, other types of endogenous hyperinsulinemia are likely [3]. Therefore, this test further confirmed the possibility of insulin autoantibody syndrome in this patient.

Insulin autoantibody levels were checked, which took a period of 1 month, as the sample was sent to India for testing; the level amounted to > 300 U/mL (< 12), and thus the patient was diagnosed with insulin autoantibody syndrome. He was commenced on prednisolone 30 mg mane. His hypoglycemic episodes responded dramatically to the steroids, leading to a significant improvement of his symptoms, and he was gradually tapered off them.

## Discussion

The pathogenesis of IAS occurs in two phases. In response to a meal, insulin secreted by the pancreas gets bound to the insulin autoantibodies and thus becomes inactive, leading to a transient hyperglycemia. This further stimulates insulin secretion. Subsequent spontaneous dissociation of insulin from the bound complexes leads to hypoglycemic attacks. Therefore, most patients present with postprandial hypoglycemia [1]. This patient had very severe, frequent hypoglycemic episodes and a markedly elevated insulin level. Interestingly, he experienced mainly fasting hypoglycemia, indicating the need to consider IAS even when the pattern of hypoglycemia is not postprandial.

The diagnosis of IAS can be aided by the insulin/C-peptide ratio and post-PEG recovery and confirmed by insulin autoantibody levels. In a normal individual, insulin and C-peptide have a half-life of 5–10 minutes and 30–35 minutes, respectively. Therefore, the insulin/C-peptide ratio will be less than 1 as insulin degrades faster. However, in IAS, as insulin is bound in insulin–insulin autoantibody complexes, a prolonged half-life will be present, resulting in an insulin/C-peptide ratio of more than 1. The recovery of insulin levels when the supernatant is tested after precipitation with PEG is significantly lower in patients with IAS than healthy individuals. Thus, with these supportive investigations, insulin autoantibody levels can be tested [1].

With regard to a possible trigger, alpha-lipoic acid and methimazole are the well-recognized drugs that trigger IAS. Literature postulating the possibility of a multitude of drugs such as clopidogrel, gliclazide, diclofenac, and omeprazole causing IAS are present; however, the strength of evidence is low [1]. The timing of drug-induced IAS is reported to be diverse, from within few days up to many months. Two case reports describe IAS possibly caused by alpha-lipoic acid in which symptoms developed within 2 weeks of starting the supplement [4, 5]. A case series of six patients with IAS caused by alpha lipoic acid reports a symptom onset of 30–120 days after taking lipoic acid [3]. IAS caused by methimazole was reported in a patient with Graves’ disease in which onset of hypoglycemic attacks was after 4 weeks [6]. Mean onset has been estimated as 4–6 weeks from the onset of drug therapy [7]; however, reports that detail the onset of IAS years after the first administration of the drug have also been reported. A case report describes a patient Graves’ disease who had been treated with methimazole 2 years prior to the onset of hypoglycemic attacks. In this case the attacks had begun after the third course of methimazole therapy [8].

Coriander and ginger are spices that are known to cause hypoglycemia. A study done in 1998 to assess the mechanism of hypoglycemia in coriander found that supplementation of 10% coriander seeds led to marked hypoglycemia in rats. This was postulated to be due to increased glycolysis and decreased gluconeogenesis. This study further reported the possibility of hypoglycemia due to increased insulin, by the stimulation of pancreatic secretion or by activation of the inactive form [9].

A study conducted on diabetic rats reported that ginger extract produced a significantly high insulin levels and decreased fasting blood glucose [10]. Another study compared the effects of ginger extracts in diabetic and normal rats and reported a reduction of fasting blood glucose in diabetic rats to the normal range [11]. A similar effect has also been reported with raw ginger administered to diabetic mice [12]. The continuous daily use of an herbal supplement containing these ingredients for a period of over a year makes consideration of a possible association with IAS worthwhile. However, it should be noted that the supplement had been consumed 2 years prior to the onset of symptoms.

A temporal relationship exists between the use of omeprazole and the onset of symptoms, with an interval of approximately 4 weeks. A case report of omeprazole-induced IAS was published in 2016, where a 65-year-old woman presented with recurrent hypoglycemic attacks. In this case report the hypoglycemic episodes significantly reduced following cessation of omeprazole therapy with no specific treatment with corticosteroids. However, it should be noted that the levels of anti-insulin antibodies in this patient were > 45 U/mL, whereas our patient had levels more than 300 U/mL [13].

Another case of IAS caused by esomeprazole was reported in 2022 in China in a 58-year-old man who was commenced on the drug 6 weeks prior to the onset of symptoms. In this case, too, symptoms improved following discontinuation of the drug, but similar to the previous case report, the anti-insulin antibody level was > 50 U/mL [14].

The temporal relationship makes omeprazole-induced IAS the likely possibility in this patient.

## Conclusion

IAS should be considered as a differential diagnosis even in patients presenting with fasting hypoglycemia, and this case report sheds light on the link between the common over-the-counter drug omeprazole and insulin autoantibody syndrome. Furthermore, the known hypoglycemic effects of coriander and ginger make it worthwhile to consider a possible association of these spices with IAS.

## Data Availability

Not applicable.

## Abbreviations

IAS: Insulin autoantibody syndrome
PEG: Polyethylene glycol
WBC: White blood cell
TSH: Thyroid-stimulating hormone
CECT: Contrast-enhanced computed tomography
